# Effects of IGF-1 on the Three-Dimensional Culture of Ovarian Preantral Follicles and Superovulation Rates in Mice

**DOI:** 10.3390/biology11060833

**Published:** 2022-05-29

**Authors:** Shizhen Dai, Hanxue Zhang, Feng Yang, Wei Shang, Shenming Zeng

**Affiliations:** 1 National Engineering Laboratory for Animal Breeding, Key Laboratory of Animal Genetics and Breeding of the Ministry of Agriculture, College of Animal Science and Technology, China Agricultural University, Beijing 100193, China; b20183040312@cau.edu.cn (S.D.); hanxuezhang@cau.edu.cn (H.Z.); yangcfeng@cau.edu.cn (F.Y.); 2 Department of Obstetrics and Gynecology, Chinese PLA General Hospital, Center for Reproductive Medicine, The Sixth Medical Center, Beijing 100037, China

**Keywords:** IGF-1, follicle development, three-dimensional culture, mouse

## Abstract

**Simple Summary:**

In this experiment, mouse secondary follicles were cultured in the three-dimensional culture system of alginate gel with different concentrations of IGF-1. According to the results of follicle growth, oocyte maturation, the levels of hormone (17β-estradiol, progesterone and AMH) and the expressions of genes related to hormone secretion, oocyte-secreted factors, apoptosis and gonadotropin receptor, the optimal concentration of IGF-1 was determined to be 10 ng/mL in the culture medium. Moreover, the intraperitoneal injection of IGF-1 before superovulation in mice could increase the number of ovulated oocytes and reduce their degraded rates.

**Abstract:**

Insulin-like growth factor-1 (IGF-1) plays a crucial role during folliculogenesis, which has been demonstrated by previous research. However, the optimal IGF-1 dosage in the three-dimensional (3D) culture system is unknown. Mouse secondary follicles (140–150 µm) were cultured for 6 days within an alginate bead in a medium supplemented with 0 (G0), 5 ng/mL (G5), 10 ng/mL (G10), or 50 ng/mL IGF-1 (G50). Secretions of 17β-estradiol and progesterone were significantly increased in G10 and G50 (*p* < 0.05). However, G50 significantly inhibited follicular growth (*p* < 0.05), while G10 showed a higher oocyte maturation rate. Thus, the 10 ng/mL IGF-1 was used in subsequent experiments. IGF-1 enhanced the function of granulosa cells (GCs) by upregulating expressions of *Star*, *Cyp19a1*, *Hsd3b1*, *Fshr*, and *Lhcgr*. Oocyte secretory function was promoted by upregulating expressions of *Bmp-15,* *Gdf-9*, and *Fgf-8*. Addition of IGF-1 showed anti-apoptotic effect. However, G10 did not improve fertilization rate of MII oocytes compared to G0. In an intraperitoneal injection experiment in mice, IGF-1 significantly increased the number of ovulated oocytes *(p <* 0.05). In conclusion, 10 ng/mL IGF-1 can promote the production of mature oocytes in the 3D culture medium and injection of IGF-1 before superovulation increases the number of ovulated oocytes.

## 1. Introduction

Follicular development is gonadotropin-independent at the earliest growth stage [1] when it is mainly regulated by paracrine or endocrine factors [2]. Insulin-like growth factor-1 (IGF-1) regulates many intraovarian activities (follicular growth, hormones selection, atresia, cell differentiation, steroidogenesis, and oocyte maturation) as a paracrine or endocrine factor during follicular development [3] and performs its biological function by binding to IGF binding proteins (IGFBPs) or IGF-1 receptor (IGF-1R) [4]. Previous research reported that *Igf-1* was expressed in the ovaries of different species [5,6], and IGF-1 could improve oocyte maturation rate and quality in vitro [7]. Both male and female *Igf-1* knockout mice were infertile, and the size of the ovaries of *Igf-1*^−/−^ female mice was less than half of the normal. Furthermore, most follicles were mostly arrested in the preantral stage, while a few follicles developed into the antral stage without maturation and ovulation [8]. The transcripts of *Igf-1* are low during the primary follicle stage and reach a high level in the late preantral and early antral stages in mice [9]. In addition, the development of mouse early secondary follicles with diameters less than 150 µm was promoted by the addition of IGF-1 [5]. These results indicate that the regulatory function is concentrated at the rapid development stage from the secondary follicles to antral follicles.

In rats, IGF-1 and IGFR are located in oocytes during the preantral follicle stage and the intensity of IGFR is stronger than IGF-1 based on immunohistochemistry analysis [10]. The oocyte is the main source and site of action, and similar results appeared in a study on sheep [11]. These previous studies showed that the endogenous paracrine system of IGF-I plays an important role and the oocyte was a site of action during the preantral follicle stage. The oocyte–granulosa cell interaction during the early follicular growth stage has been indicated [12]. In vitro studies have shown that IGF-1 also can prevent granulosa cells (GCs) apoptosis and enhance proliferation [3] which showed that the regulation of GCs by IGF-1 may be mediated by oocytes. The expression of *Fshr* in preantral follicles of IGF-1^−/−^ mice remained at a low level, while the IGF-I can significantly increase *Fshr* expression in murine granulosa cells in vivo [13]. The effect of IGF-1 on the differentiation and proliferation of GCs may be related to gonadotropin receptors.

Excessive concentrations of IGF-1 can upregulate apoptosis-regulating genes, increase the vulnerability of GCs and lead to apoptosis [14]. Yang et al., showed that 10 ng/mL of IGF-1 treatment inhibited apoptosis in cultured bovine GCs, while higher concentrations of IGF-1 (100 ng/mL) promoted apoptosis [15]. These results indicate that the role of IGF-1 in inhibiting apoptosis is dose-dependent. Similarly, mouse preantral follicles in traditional in vitro culture exposed to 50 ng/mL IGF-1 significantly increased estradiol secretion at 4 day [16]. This implied that the effect of IGF-1 on follicular development is dependent on its dosage.

Maintaining follicular structure during the culture period facilitates the gap junction between the oocyte and the GCs, which is essential for oocyte growth and meiotic competence [17]. The disruption of follicle structure by two-dimensional culture can be solved by three-dimensional (3D) culture. The 3D culture system provides a physical force to maintain normal morphologies of follicles and ensures a normal paracrine environment [18]. To mimic an in vivo environment, a variety of 3D culture systems have been developed and many studies have been reported [19,20]. There are several types of matrices [18] (collagen, alginate, and hyaluronan hydrogel [21]). The advantage of an alginate hydrogel over other substances is that a solution containing the binding agent (Ca^2+^) can be added to complete polymerization [22]. This property allows the follicle to be easily embedded under physiological conditions [23]. In the sodium alginate culture system, mouse, monkey, or human secondary follicles can develop normally [24]. Optimized follicular culture systems promote the production of high-quality oocytes, which have been successfully applied in various animal models, including humans and non-human primates, and have produced offspring in rodents [25].

In this study, we determined the optimal concentration of IGF-1 in a 3D in vitro culture system. The function of IGF-1 was analyzed by the changes in hormone secretion and gene expression and the synergistic effect of IGF-1 and gonadotropin was demonstrated in vivo during superovulation.

## 2. Materials and Methods

### 2.1. Media and Chemicals

MEM α with GlutaMAX^™^, Ham’s F-12 Nutrient Mixture, Leibovitz’s L-15, Collagenase IV, and fetal bovine serum (FBS) were purchased from Gibco (Gibco BRL, Grand Island, NY, USA). Other chemicals were obtained from Sigma-Aldrich (Sigma-Aldrich Co. LLC, St. Louis, MO, USA) unless otherwise specifically indicated.

### 2.2. Animals

All animals were purchased from SPF (Beijing, China) Biotechnology Co., Ltd. All mice were housed in polypropylene cages and provided food and water ad libitum. Animals were kept on a 12 h light/dark cycle (8:00 a.m.–8:00 p.m.) at 22–25 °C with 30–50% relative humidity.

### 2.3. Follicle Isolation, Encapsulation, and Culture

Ovaries isolated from 16 days old ICR-1 female mice were divided into two pieces and placed in enzymatic media containing L-15 with 0.2% Collagenase IV and 0.2% DNase I for 40 min at 37 °C before follicle isolation. Secondary follicles (140–150 µm) with theca cells were then mechanically isolated using insulin-gauge needles in L-15 media with 1.0% FBS and encapsulated into a sterile 0.5% (*w*/*v*) alginate bead. Beads were incubated in half MEM α and half F-12 of the mixture containing 10% FBS at 37 °C, 5% for 30 min. Then, beads were plated (one follicle/well) in 96-well plates in 100 µL of culture media composed of MEM α, 3 mg/mL of BSA, 1 mg/mL bovine fetuin, 5 µg/mL of insulin, 5 µg/mL of transferrin, and 5 ng/mL of selenium. Encapsulated follicles were cultured at 37 °C in 5% CO_2_ for 6 days. Every other day, half of the media (50 µL) was exchanged and stored at −80 °C. Follicle survival and diameter were assessed using an inverted microscope (Olympus IX-70) with transmitted light and phase objectives. Follicles were designated dead if the oocyte was no longer surrounded by a granulosa cell layer or if the GCs had become dark and fragmented.

After 6 days, the culture medium was replaced by 100 µL L-15 medium containing 10 mIU/mL alginate lyase for 30 min at 37 °C.

### 2.4. Follicle Diameter Measurement

Pictures of encapsulated follicles were taken on culture day 0, 2, 4, and 6 using an inverted microscope with the same setup parameters. The diameters of follicles were measured in duplicate from the outer layer of theca cells by Image J.

### 2.5. Oocyte In Vitro Maturation

After follicles >300 μm were retrieved from the alginate bead, they were transferred to a maturation media composed of MEM α, 10% FBS, 3 IU/mL human chorionic gonadotropin (hCG), 10 ng/mL epidermal growth factor (EGF), and 10 mIU/mL porcine FSH (SIOUX Biochemical, Inc., Sioux Center, IA, USA) for 16 h at 37 °C, 5% CO_2_. Oocytes were then denuded from the surrounding cumulus cells by treatment with 0.3% hyaluronidase and gentle aspiration through a polished drawn glass pipette. The oocytes were considered to be in metaphase I if neither the germinal vesicle nor the first polar body was visible. If a polar body was present in the perivitelline space, the oocytes were classified as metaphase II. Fragmented or shrunken oocytes were classified as degeneration and were discarded.

### 2.6. Oocyte In Vitro Fertilization

Sperm was collected from the cauda epididymis of 9-week-old ICR-1 male mice. One hour before IVF, motile sperm was incubated in 100 μL mHTF medium (Irvine Scientific, Santa Ana, CA, USA) under mineral oil at 37 °C, 5% CO_2_. Twenty metaphase II oocytes were placed in 50 μL mHTF medium drops containing 10^6^ sperm/mL and incubated under mineral oil for 6 h at 37 °C, 5% CO_2_. Oocytes were then washed 3 times in fresh G1-PLUS^TM^ (Vitrolife, Göteborg, Sweden) medium to remove all bound sperm and cultured in G1-PLUS^TM^ medium at 37 °C, 5% CO_2_. Fertilized oocytes were identified by the presence of 2 pronuclei (2PN).

### 2.7. Immunostaining and Confocal Imaging

For immunostaining, oocytes were fixed in 4% paraformaldehyde at 4 °C for 60 min. The oocytes were washed three times in PBS containing 0.1% PVA and transferred to 3% bovine serum albumin (BSA) at 37 °C for 60 min. Blocked oocytes were incubated at 37 °C with CoraLite594-Conjugated Alpha Tubulin Monoclonal Antibody (1:200, CL594-66031, Proteintech, Thermo Fisher Scientific, Waltham, MA, USA) for 60 min in the dark. After washing three times with PBS containing 0.1% PVA, chromosomes were labeled using Hoechst 33342 for 5 min and oocytes were mounted in 2.5 μL Antifade Mounting Medium drops on glass bottomed dishes. Samples were examined under a confocal laser scanning microscope.

### 2.8. Assay of 17β-Estradiol, Progesterone, and AMH

The hormone determination work was completed by the Beijing JinHaiKeYu Biological Technology Development Co. Ltd. (JinHaiKeYu Biological Technology Development Co. Ltd., Beijing, China). The 17β-estradiol and progesterone in conditioned media were measured using a radioimmunoassay. AMH in conditioned media was determined by ELISA. The sensitivities for the 17β-estradiol, progesterone and AMH assays were 5 pg/mL, 30 pg/mL, and 10 pg/mL. Intraassay and interassay coefficients of variation were determined to be 10 and 15%, respectively. To obtain sufficient media for each assay, media collected from follicles in identical alginate conditions were pooled for each time point (10 samples pooled per measurement). Five independent measurements for each hormone at each time point were performed.

### 2.9. Characterization of Genes Expression

Total RNAs from the follicles were extracted and purified using the RNeasy Micro Kit (TransGene, Beijing, China). The total RNAs were reverse transcribed by Maxima^TM^ H Minus cDNA Synthesis Master Mix (Thermo Fisher Scientific, Waltham, MA, USA) and the first-strand cDNAs were used for Q-PCR analysis with GoTaq^®^ qPCR Master Mix (Promega, Madison, WI, USA). The primer sequences used are listed in Table 1. We have performed the melting curve and the quality of primers are very good, and no dimers are formed.

Real-time quantitative polymerase chain reaction (qPCR) was performed using the qTOWER 2.0/2.2 sequence detection system (Analytik Jena, Jena, TH, Germany). For each reaction, 2 μL cDNA, 0.4 μL primers, 10 μL 2×Master SYBR Green mix (Promega), and 7.2 μL nuclease-free water were added to a final volume of 20 μL. PCR cycling conditions were 95 °C for 15 min, followed by 39 amplification cycles of 95 °C for 10 s, 60 °C for 20 s, and 72 °C for 15 s. After testing, the efficiency of qPCR is between 95–98%. To maximize accuracy, each sample was run in triplicate with a negative control of the reaction mixture with no cDNA added.

### 2.10. Injection Protocol of IGF-1 during Mouse Superovulation

**Figure 1:**
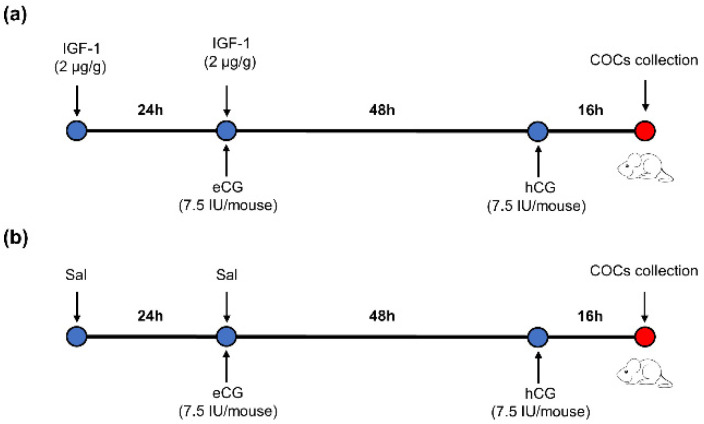
The protocol of IGF-1 administration during mouse superovulation. (**a**) IGF-1 treatment group; (**b**) Control group. 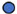: Time of injection. 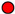: Time of mice euthanasia by cervical dislocation.

Sixty ICR-1 8-week-old female mice were randomly divided into two groups. There were 3 repetitions per group and 10 mice per repetition. The mice were injected intraperitoneally with IGF-1 (2 μg/g body weight per mouse) 24 h before equine chorionic gonadotropin (eCG) (7.5 IU per mouse) injection, followed by injection with hCG (7.5 IU per mouse) 48 h after eCG injection in the IGF-1 treatment group. Mature metaphase II oocytes were collected from the oviducts 16 h after hCG injection (Figure 1a). The control group mice were injected with the same dosage of saline (Sal) as IGF-1 at the same time point (Figure 1b).

### 2.11. Statistical Analysis

Experimental data were obtained from three or more independent repeated experiments. Percentage data were arcsine transformed. Differences in follicular diameter, survival rate, maturation rate, degeneration rate, hormonal production, and the number of ovulated oocytes were analyzed by one-way ANOVA (SAS, Cary, NC, USA). The expression level of different genes was analyzed using Student’s *t*-test. The percentages of normal and degraded oocytes were analyzed by chi-square analysis. Data were reported as mean ± SD. *p* < 0.05 * was considered statistically significant. All statistical calculations were performed using SAS (version 9.0) software (Cary, NC, USA).

## 3. Results

### 3.1. Effects of IGF-1 on Follicular Development and Maturation In Vitro

**Figure 2:**
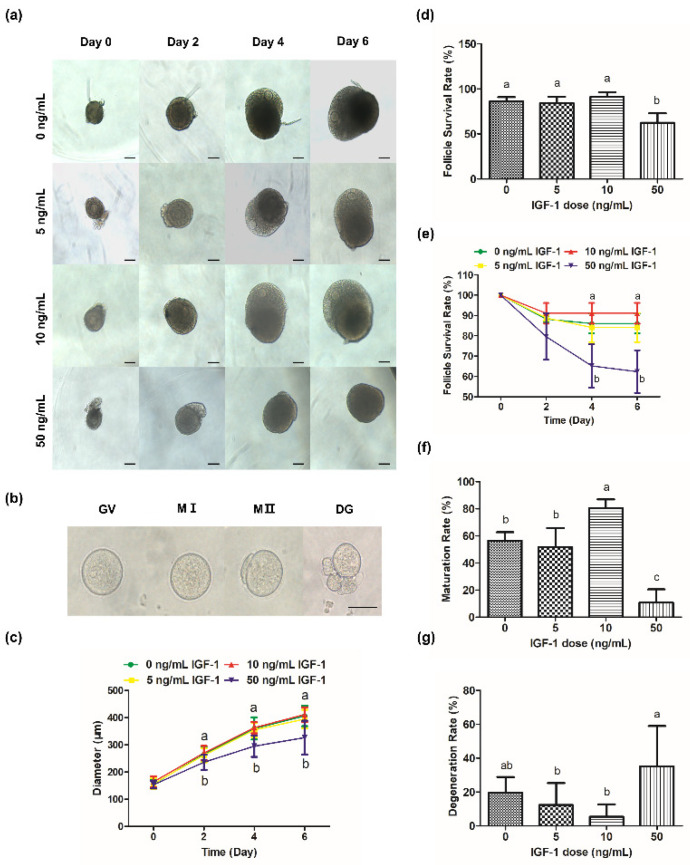
Effects of IGF-1 on mouse follicle growth and oocyte maturation. (**a**) Images of follicular growth in media containing different concentrations of IGF-1 (0, 5, 10, and 50 ng/mL) from day 0 to day 6; (**b**) Images of GV, MI, MII, and DG (degeneration) oocytes after IVM; (**c**) Line graph of change in follicle diameters in media containing different concentrations of IGF-1 from day 0 to day 6; (**d**) Follicle survival rates at day 6 in media containing different concentrations of IGF-1; (**e**) Line graph of change in follicle survival rates in media containing different concentrations of IGF-1 from day 0 to day 6; The maturation (**f**) and degeneration (**g**) rates of oocytes after IVM were isolated from follicles treated with different concentrations IGF-1 for 6 days. Scale bar in (**a**): 100 μm; (**b**): 50 μm. Error bar: standard deviation; points or bars with completely different letters (a, b, c) indicating significant difference among treatments for isolated time points (*p* < 0.05). *n* = 10 follicles for five replicates.

During culture processing, the follicles of the control group increased from 156.21 ± 16.85 μm at 0 day to 406.14 ± 37.74 μm at 6 day (Figure 2c) and produced mature oocytes with meiotic ability after mature culture (Figure 2b). The oocytes were visible and the follicles were transparent during culture in G0, G5, and G10 (Figure 2a). However, GCs became darker and the edges of the oocytes were blurred or invisible (Figure 2a), and the survival rate was significantly reduced in G50 at 4 day and 6 day (*p* < 0.05) (Figure 2d,e). Meanwhile, there was a significant decrease in follicular size in G50 on day 2 compared with the other groups (*p* < 0.05) (Figure 2c). However, there was no significant change in follicular size in G5 and G10 compared with the control group (*p* > 0.05) (Figure 2c).

Compared with the maturation rate of the G0, G10 was significantly increased (*p* < 0.05) while G50 was significantly decreased (*p* < 0.05) (Figure 2f). Meanwhile, there was no significant difference in the maturation rate of G5 compared with the G0 (*p* > 0.05) (Figure 2f). There was no significant difference in the degeneration rate of G5 and G10 compared with G0 (*p* > 0.05) (Figure 2g). However, the degeneration rate of G50 was the highest (32.14%), which was significantly higher than G5 and G10 (*p* < 0.05) (Figure 2g).

### 3.2. Effects of IGF-1 on 17β-Estradiol, Progesterone, and AMH Secreted by Follicles Cultured In Vitro

**Figure 3:**
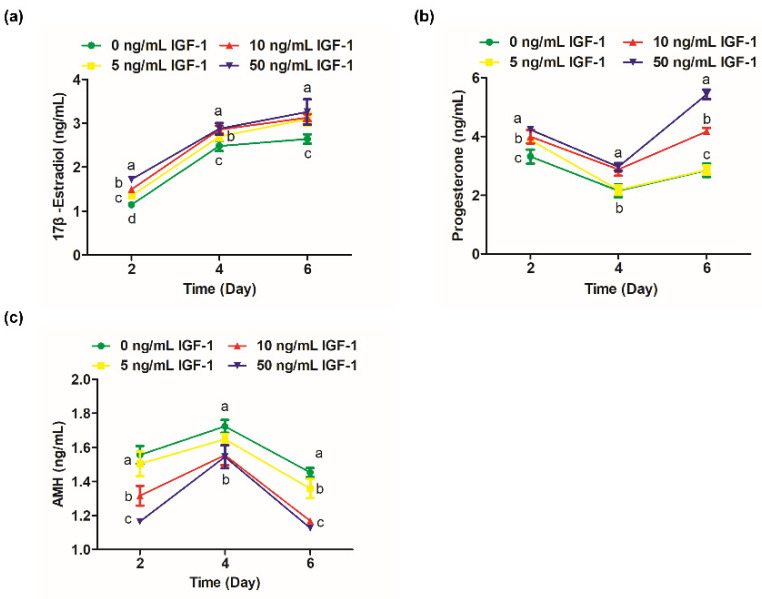
Effect of IGF-1 on hormone secretion of in vitro cultured follicles. (**a**) The secretion of 17β-estradiol; (**b**) progesterone and (**c**) AMH at days 2, 4, and 6. Error bar: standard deviation; points with completely different letters (a, b, c) indicating significant difference among treatments for isolated time points (*p* < 0.05). *n* = 10 follicles for three replicates.

The 17β-estradiol significantly increased from days 2 to 4 of culture (Figure 3a). On day 2, the secretion of 17β-estradiol in G0 was significantly lower than the other groups (*p* < 0.05), and the level of 17β-estradiol was positively correlated with the dosage of IGF-1 (Figure 3a). On day 4, the 17β-estradiol levels in G10 and G50 converged and were significantly higher than G0 and G5 (*p* < 0.05) (Figure 3a). However, the difference between the treatment groups was no longer significant by day 6 (*p* > 0.05), but still significantly increased the amount of 17β-estradiol secretion compared with the G0 (*p* < 0.05) (Figure 3a). Progesterone levels decreased from day 2 to day 4 and increased from day 4 to day 6. On day 2, the progesterone level in G0 was significantly lower than the treatment groups (*p* < 0.05) (Figure 3b). On day 4, the progesterone level in G5 was not significantly different from the G0 (*p* > 0.05) and was maintained until the end of the culture (Figure 3b). The progesterone levels of G10 and G50 were at a high level, and there was no significant difference on day 4 (*p* > 0.05) (Figure 3b). However, there was a significant difference at day 6 when G50 significantly increased the secretion of progesterone compared to the other groups (*p* < 0.05) (Figure 3b). AMH, secreted by the growing follicles, was detectable by day 2 and peaked at day 4 (Figure 3c). The G10 and G50 significantly decreased the secretion of follicular AMH on days 2, 4, and 6 in comparison to the other groups (*p* < 0.05) (Figure 3c). The amount of AMH secreted was also reduced in G5 on day 6 compared with the G0 (*p* < 0.05) (Figure 3c).

### 3.3. Effect of 10 ng/mL IGF-1 on the Expression of Hormone Secretion Related Genes, Oocyte-Secreted Factors Genes, and Gonadotropin Receptor Genes In Vitro

**Figure 4:**
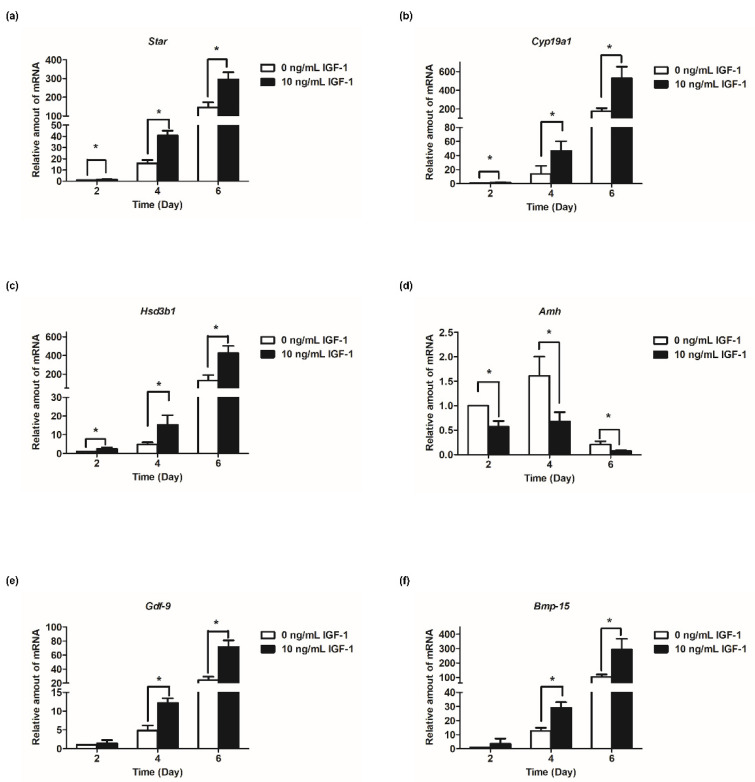

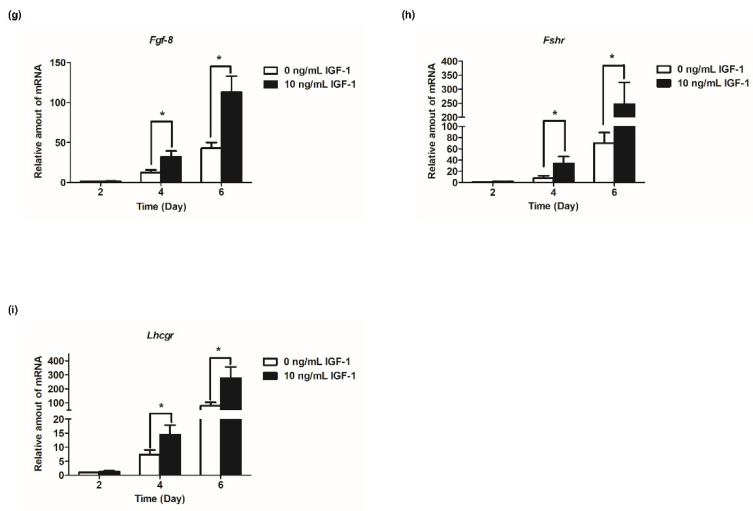
Effect of IGF-1 on the mRNA expression of hormone secretion related genes, oocyte-secreted factors and gonadotropin receptors in vitro. (**a**) The mRNA expression of *Star*; (**b**) The mRNA expression of *Cyp19a1*; (**c**) The mRNA expression of *Hsd3b1*; (**d**) The mRNA expression of *Amh*; (**e**) The mRNA expression of *Gdf-9*; (**f**) The mRNA expression of *Bmp-15*; (**g**) The mRNA expression of *Fgf-8*; (**h**) The mRNA expression of *Fshr*; and (**i**) The mRNA expression of *Lhcgr*. * *p* < 0.05 compared to the control group; error bar: standard deviation. *n* = 10 follicles for each of three replicates.

Based on the results of follicular development and hormone secretion, the best concentration of IGF-1 was 10 ng/mL. Subsequently, the mRNA transcript levels of hormone secretion-related genes were measured. The results showed that the expression of steroid synthesis-related genes (*Star*, *Cyp19a1*, and *Hsd3b1*) were increased with the development of follicles, and IGF-1 treatment significantly increased the expression level (*p* < 0.05) in all stages of in vitro culture of follicles (Figure 4a–c). The transcriptional and expression level of *Amh* mRNA increased from days 2 to 4, but from day 4 to day 6, the expression level decreased (Figure 4d). At all stages of culture, IGF-1 significantly downregulated the expression level of *Amh* mRNA (*p* < 0.05) (Figure 4d). *Gdf-9, Bmp-15*, and *Fgf-8* are expressed at all stages of in vitro follicular development and their expression increased tens or even hundreds of times as the number of days of culture increased, reaching its highest at day 6 (Figure 4e–g). On day 4, 10 ng/mL IGF-1 treatment had significantly upregulated the expression of these three genes (*p* < 0.05), *Gdf-9* (D4: 2.54-fold; D6: 2.94-fold) (Figure 4e), *Bmp-15* (D4: 2.30-fold; D6: 2.86-fold) (Figure 4f), and *Fgf-8* (D4: 2.62-fold; D6: 2.63-fold) (Figure 4g). The gonadotropin receptor genes *Lhcgr* and *Fshr* also increased with the growth and development of follicles and, from day 4, the expression level of the treatment group was significantly higher than that of the control group (*p* < 0.05) (Figure 4h,i). On day 4, *Fshr* mRNA expression was upregulated by 4.45-fold (Figure 4h), and by day 6 *Lhcgr* mRNA expression was upregulated by 3.49-fold (Figure 4i).

### 3.4. Effect of IGF-1 on the Expression of Apoptosis-Related Genes In Vitro

**Figure 5:**
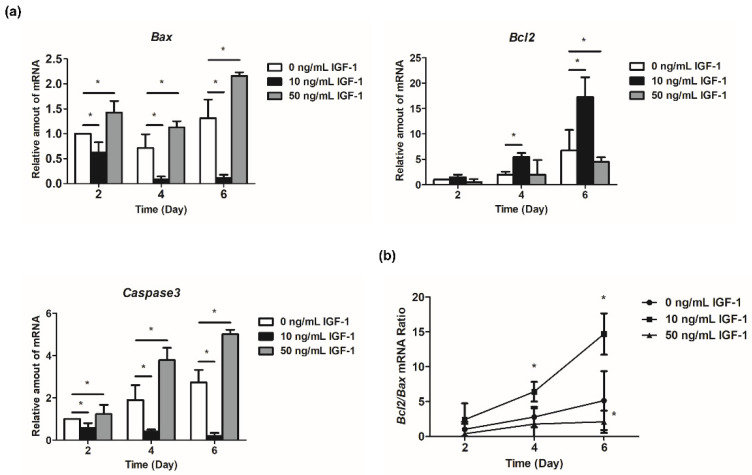
Effect of IGF-1 on apoptosis-related gene expression in vitro. (**a**) The relative expression level of *Bcl2*, *Bax*, and *Caspase3*; (**b**) The *Bcl2/Bax* mRNA ratio. * *p* < 0.05 compared to control group; error bar: standard deviation. *n* = 10 follicles for three replicates.

The expression of *Bcl2* increased with the development of follicles (Figure 5a). The regulation of 10 ng/mL IGF-1 reached a significant level at day 4 and day 6, which increased the expression level of *Bcl2* in the treatment group (*p* < 0.05) (Figure 5a). Additionally, 50 ng/mL IGF-1 significantly decreased the expression level of *Bcl2* compared with the G0 at day 6 (*p* < 0.05) (Figure 5a). *Bax* initially decreased and then increased during in vitro follicular development (Figure 5a). The addition of 10 ng/mL IGF-1 significantly downregulated its expression at each stage, while 50 ng/mL IGF-1 significantly increased its expression at each stage (*p* < 0.05) (Figure 5a). Furthermore, 10 ng/mL IGF-1 treatment also significantly increased the *Bcl2/Bax* ratio after 4 days of culture (*p* < 0.05) (Figure 5b). Meanwhile, 50 ng/mL IGF-1 treatment significantly decreased the *Bcl2/Bax* ratio at day 6 (*p* < 0.05) (Figure 5b). The expression level of *Caspase3* increased with the development of follicles in G0, and the addition of 10 ng/mL IGF-1 significantly inhibited its expression in follicles (*p* < 0.05) (Figure 5a). This trend was opposite to the G0, with decreased follicular development (Figure 5a). Finally, 50 ng/mL IGF-1 significantly increased the expression level of *Caspase3* compared with the G0 at each stage (*p* < 0.05) (Figure 5a).

### 3.5. Effect of 10 ng/mL IGF-1 on Oocyte Quality and Fertilization Rate In Vitro

**Figure 6:**
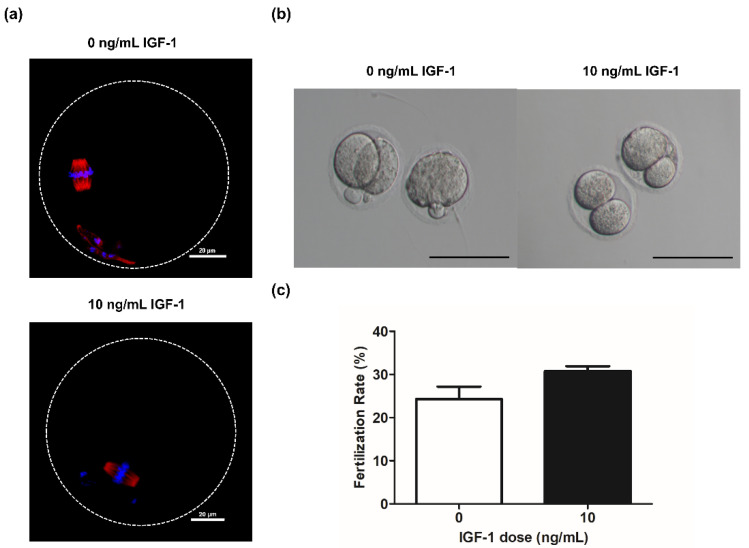
(**a**) Grown oocytes underwent germinal vesicle breakdown and progressed to metaphase II in group G0 and G10; (**b**) Embryo morphology at the two-cell stage in group G0 and G10. Scale bar: 100 μm; (**c**) The fertilization rate in group G0 (*n* = 37) and G10 (*n* = 39).

Spindle formation and chromosome alignment are important for the competence of oocytes. Accordingly, we evaluated the effect of adding IGF-1 on these parameters during in vitro culture. The results showed that the oocytes obtained from the G0 and G10 groups were able to form normal spindle and maintain normal chromosome distribution after in vitro maturation (Figure 6a). Meanwhile, there was no significant difference in the fertilization rate of G10 compared with G0 (12/39 vs. 9/37, *p* > 0.05) (Figure 6c). The oocytes in both G0 and G10 can develop to the two-cell stage after in vitro fertilization (Figure 6b).

### 3.6. Effect of IGF-1 Treatment on Oocyte Number and Quality during Superovulation

**Figure 7:**
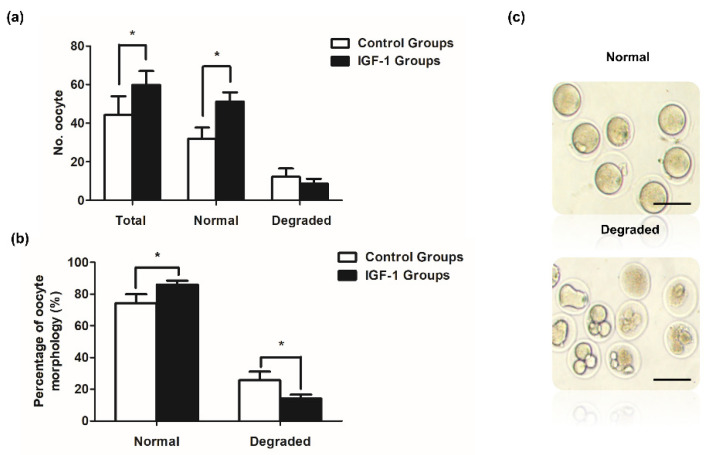
Ovulated oocytes in vivo in mice treated with IGF-1 (*n* = 30) or saline control (*n* = 30). (**a**) The in vivo number of ovulation oocytes, normal oocytes, and degraded oocytes of mice in IGF-1 treated group or control group; (**b**) Rates of normal oocytes and degraded oocytes of mice in vivo in the IGF-1 treated group or control group; and (**c**) The morphology of normal oocytes and degraded oocytes. Scale bar: 100 μm. * *p* < 0.05 compared to control group.

Mice in the control and IGF-1 treated groups exhibited significant differences. Both the number of ovulated oocytes and normal oocytes were significantly higher in the IGF-1 treated group than in the control group *(p* < 0.05) (Figure 7a). The number of degraded oocytes was lower in the IGF-1 treated group compared to the control group (Figure 7a). Figure 7b shows that the rate of normal oocyte morphology was significantly higher than the control group after injecting IGF-1 (1403/1639 vs. 938/1302, *p* < 0.05), and the IGF-1 treated group had a significantly decreased percentage of degraded oocytes (236/1639 vs. 364/1302, *p* < 0.05).

## 4. Discussion

The 3D culture system could maintain the structure of follicles and the gap junction between cells. We systematically studied the regulation of IGF-1 to whole follicles, especially the effect on the related functional genes of oocytes and granulosa cells. Furthermore, according to the results of in vitro experiments, this has also been verified in vivo. Previous in vitro studies have revealed the growth-promoting effect of IGF-1 in different species [26,27,28]. Our results showed that 10 ng/mL IGF-1 increased the mouse follicle diameter compared to the control (Figure 2c). It also significantly improved the ability of oocyte meiosis resumption (*p* < 0.05) (Figure 2f), while 50 ng/mL IGF-1 significantly inhibited follicular growth (*p* < 0.05) (Figure 2a,c). There was no significant difference in the subsequent fertilization rate of mature oocytes obtained through in vitro maturation under different treatment conditions [16], which was the same in our study. IGF-1 can directly influence ovarian function, and the effects depend on the concentration. High concentrations of IGF-1 may destroy the function of preantral follicles. Previous results showed that the ultrastructural features of follicles were normal in the presence of 30 ng/mL IGF-1 in vitro. In contrast, follicles displayed signals of degeneration at a 100 ng/mL concentration [29]. High concentrations of IGF-1 (100 ng/mL) also promoted bovine GCs apoptosis [15]. These results suggest that the effects of IGF-1 on follicular development are dose-dependent. Our results showed that the 10 ng/mL and 50 ng/mL IGF-1 promoted steroidogenesis at all stages (Figure 3a,b). Similar results have been found in other studies [16].

In hormone production, our results showed that IGF-1 had a promotional effect on follicular steroidogenesis. Estradiol synthesis is primarily regulated by gonadotropins and IGF-1 can positively or negatively alter the concentration of estradiol produced by promoting differentiation and proliferation of GCs [30]. With the formation of antral follicles, the production of 17β-estradiol and progesterone are usually increased [31]. The curve of the diameter of follicles showed that follicles grew fastest from day 2 to day 4, which is the same trend as the 17β-estradiol secretion curve (Figure 2c and Figure 3a). IGF-1 promotes steroid production by increasing the sensitivity of GCs to FSH [32]. Similar results were found in this study. Treatment with 10 ng/mL and 50 ng/mL IGF-1 promoted progesterone production at all stages (Figure 4b). The progesterone was secreted by the theca cells during the development of follicles and the promotion effect of IGF-1 should be related to the receptor of LH on the theca cells. It can be seen that the secretion of progesterone decreased during the process of the rapid development of follicles (Figure 2c and Figure 3b). This is due to the large amount of progesterone being consumed as a precursor for the synthesis of 17β-estradiol from day 2 to day 4. There is the same trend in the culture system of buffalo preantral follicles [33]. Subsequently, the progesterone level increased from day 4 to day 6 due to the accumulation of hormone secretion. In the early stages of ovulation, higher levels of plasma progesterone help oocyte meiosis resumption and quality improvement in dairy cows [34]. In addition, the previous report has shown that adding 10 ng/mL progesterone during follicle development did not inhibit the growth of secondary follicles in the ovary [35]. Our results showed that the secretion of progesterone is less than this safe concentration. Additionally, the normal development of follicles in G10 also confirms this. Another study also had progesterone concentrations similar to ours, and their results showed that 50–72% of the oocytes produced in the 3D culture system were meiotically competent [36]. In vitro exposure of neonatal mouse ovaries to anti-Müllerian hormone (AMH) reduced the number of follicles capable of growth by 50% [37]. GCs continue to express *Amh* until the early follicle stages in mice [38]. These observations support the negative impact of endogenous AMH on the development of antral follicles. IGF-1 decreased the secretion of AMH in our studies (Figure 3c).

Considering follicular development, oocyte maturation efficiency and hormone secretion level, the optimal concentration of IGF-1 in the 3D culture of mouse follicles was 10 ng/mL. With the proliferation of GCs, the steroid secretion activity in follicles increased during the culture period. IGF-1 significantly promoted the expression of the steroidogenesis-related genes (*Star*, *Cyp19a1*, and *Hsd3b1*) at each stage. These results indicated that the synthesis increased steroid hormones by upregulating the expression of genes involved in steroid synthesis. The expression of *Star, Hsd3b1*, and *Cyp19a1* in GCs is regulated by FSH stimulation, while IGF-1 alone can increase the expression of *Star, Hsd3b1*, and *Cyp19a1* [39]. The intracellular pathway triggered by FSH and IGF-1 was mediated by different receptors and then promoted GCs to produce 17β-estradiol and progesterone. During the development of mouse follicles in vitro, the expression level of *Amh* increased in the early stage of culture and decreased in the late stage of culture. In research on rhesus macaques, the trend of *Amh* was similar to our result [40]. The addition of 10 ng/mL IGF-1 significantly down-regulated its mRNA expression level which inhibited the secretion of AMH. As a result, it attenuated the inhibition of follicle development.

Oocyte-derived factors such as BMP-15, GDF-9, and FGF-8 stimulate the proliferation and differentiation of GCs, improving the developmental capacity of oocytes during in vitro maturation [41]. The addition of GDF-9 to rodent ovarian tissue during in vitro culture can promote primary follicular development [42]. In contrast, in *Gdf-9* knockout mice, follicular development was arrested in the primary phase [43]. GDF-9 has been shown to be effective in stimulating the development of rat preantral follicles cultured in vitro [44], and it also promoted early preantral follicle growth in human ovarian cortical tissue culture [45]. During the stage from preantral follicles to antral follicles, it appeared that GDF-9 improved follicular survival by suppressing granulosa cell apoptosis [46]. *Bmp-15* homozygous mutants are completely sterile, and follicles cease to develop in the primordial stage [47]. Removal of the bovine oocyte from cumulus–oocyte complexes triggered cumulus cell apoptosis, which could be prevented by BMP-15 [48]. BMP-15 was also involved in cumulus expansion by enhancing the expression of EGF-like growth factors [49]. FGF-8 plays a mediator of the oocyte to regulate follicle cell proliferation or differentiation to a large extent [50]. Additionally, both BMP-15 and FGF-8 cooperated to promote glycolysis in cumulus cells [51]. Therefore, the oocyte-derived factors in follicles are essential for the development of follicles [41]. *Gdf-9* and *Bmp-15* are expressed in an oocyte-specific manner [52]. The previous research found that IGFR was located in oocytes [10], so IGF-1 may directly act on oocytes. The present results confirmed exogenous IGF-1 really appeared this function. In this experiment, IGF-1 could significantly promote expressions of *Gdf-9, Bmp-15*, and *Fgf-8* during the later stages of culture, which showed that IGF-1 could increase oocyte-derived paracrine signals. The previous report showed that there was a synergistic relationship between GDF-9 and BMP-15, which regulated the sensitivity of GCs to FSH [53]. These results further motivate us to study the regulation of IGF-1 on gonadotropin receptors.

Our results showed that IGF-1 significantly increased the expression levels of gonadotropin receptors (*Fshr* and *Lhcgr*) in mouse follicles from day 4 to day 6, cultured in vitro (Figure 4h,i). IGF-1 can synergize with FSH to induce differentiation of mouse GCs by modulating *Fshr* expression [16]. The primary role of the IGF system is to ensure a critical level of *Fshr* necessary for gonadotropin responsiveness, and the low expression of *Fshr* is responsible for decreased follicular growth in IGF-1 knockout mice [13]. FSH stimulates serine/threonine kinase AKT which is essential for the differentiation of GCs [54]. However, in IGF-1 knockout mice, the effect of FSH on the induction of AKT phosphorylation was reduced [55]. Therefore, GCs differentiation of IGF-1 knockout mice is inhibited, because FSH fails to stimulate AKT phosphorylation in vivo. In the IGF-1 knockout state, the induction of AKT by FSH is not sufficient to induce granulosa cell differentiation [56]. This may be due to decreased expression of *Fshr*. The expression of *Lhcgr* is one of the major markers of FSH-induced granulosa cell differentiation, and this process is also modified by many growth factors [57]. In our study, Lhcgr mRNA expression levels increased with follicular development, and IGF-1 treatment significantly promoted its expression in the late stage of follicular development. IGF-1 can synergize with FSH to increase *Lhcgr* expression in rat GCs in a dosage- and time-dependent manner [57]. In the sheep, an increase in LHCGR protein immunostaining in oocytes and granulosa cells was observed after follicles culture in a medium containing IGF-1 and FSH [11]. Thus, IGF-1 has a major effect on *Fshr* expression, which in turn enhances FSH action and leads to an increase in LHCGR protein immunostaining in GCs. Other studies have also shown that IGF-1 increases the expression of *Lhcgr* in bovine GCs [58]. The synergy of IGF-1 and gonadotropins is worthy of further exploration during the development of follicles.

The Bcl2 family plays an important role in regulating the apoptotic pathway. Among them, the pro-apoptotic protein Bax is an essential protein for inducing apoptosis, and the anti-apoptotic protein Bcl2 is an inhibitor of apoptosis [59]. This study found that 10 ng/mL IGF-1 can significantly increase the Bcl2/Bax ratio from day 4 to day 6, which is critical for inhibiting the cellular apoptotic pathway. Meanwhile, 50 ng/mL IGF-1 can significantly decrease the Bcl2/Bax ratio by day 6, which also proved that the inhibition of follicle development is related to the apoptosis of GCs. Caspase3 is a key protease located downstream of the mammalian apoptosis pathway. *Caspase3* increased with time in the development of follicles in vitro, but the addition of 10 ng/mL IGF-1 significantly inhibited its expression and decreased its mRNA expression with follicular development. Apoptosis of follicular GCs in IGF1R knockout mice was significantly increased [55]. However, excessive concentrations of IGF-1 can upregulate apoptosis-regulating genes and increase the vulnerability of GCs, leading to apoptosis [14]. The addition of 50 ng/mL IGF-1 significantly increased the expression level of *Caspase3*. This is the reason why 50 ng/mL IGF-1 inhibited follicular development and caused a large percentage of oocyte degeneration in this study. Notably, although IGF-1 promoted follicular development during in vitro culture, it did not improve the oocyte developmental competence compared with the control group. We observed normal spindle morphology in oocytes after in vitro maturation in both G0 and G10 groups, which also resulted in no significant difference in fertilization rates.

Of particular note, no reverse control was set up in the in vitro culture system of this research. The IGF system involves complex regulatory networks that operate in the ovary. The bioavailability of IGF-1 is influenced by concentrations of IGF-1R and specific IGFBPs. In addition, at least six IGFBPs have been characterized, and their affinity for IGF-1 is in the same order of magnitude as that of IGF-1R [60]. Meanwhile, IGF-1 also mediated its actions through the IR [61]. We believed that complete inhibition of IGF-1 in our experiments may require more inhibitors, which may complicate the culture system. Moreover, some reported results showed that IGF-1R was expressed in granulosa cells of mammalian primary follicles [62,63], but in our experiments, the secondary follicles were used for culture in vitro, and the level of IGF-1R was high at this stage, so interference of *Igf-1r* expression by siRNA may have limited effect [3]. For the above reasons, we did not perform reverse control for IGF-1 in the culture system in vitro.

Previous reports indicated that IGF-1 can act synergistically with FSH to enhance follicular development [64]. During superovulation, equine chorionic gonadotropin (eCG) is used to stimulate follicular development, which has the same effect as FSH. In the subsequent experiment, we selected the mouse superovulation model to examine the synergistic effect of IGF-1 and gonadotropins in vivo. The results demonstrated that IGF-1 significantly increased the number of ovulated oocytes and normal oocytes during superovulation (*p* < 0.05). We suggest that IGF-1 increased the function of GCs and steroidogenesis, and upregulated the expression of gonadotropin receptors. IGF-I mediates the effect of FSH on the ovary and improves follicular development.

## 5. Conclusions

The results of the current research have proven that designing modern and powerful approaches to the murine ex vivo 3D model of IGF-1-assisted growth and maturation of ovarian secondary preantral follicles in order to obtain satisfactory outcomes of IVM might be reliable and feasible for the purposes of achieving high efficiency rates in such novel ARTs as IVF/ICSI in humans and other mammalian species [65,66,67,68] or somatic cell cloning in other mammalian species [69,70,71,72].

## Figures and Tables

**Table 1:** RT-PCR primers of target genes. Note: 39 amplification cycles of 95 °C for 10 s, 60 °C for 20 s, and 72 °C for 15 s. The efficiency of qPCR is between 95–98%.

Gene	Sequence	bp	No. NCBI
*Gapdh*	F: 5′-GGGTCCCAGCTTAGGTTCATC-3′ R: 5′-CCCAATACGGCCAAATCCGT-3′	100	NM_001289726.1
*Gdf-9*	F: 5′-TCACCTCTACAATACCGTCCGG-3′ R: 5′-GAGCAAGTGTTCCATGGCAGTC-3′	139	XM_006532220.3
*Bmp-15*	F: 5′-GCACGATTGGAGCGAAAATG-3′ R: 5′-CGTACGCTACCTGGTTTGATGC-3′	123	NM_009757.5
*Fgf-8*	F:5′-CAGGTCTCTACATCTGCATGAACAA-3′ R: 5′-TCTCCAGCACGATCTCTGTGAATA-3′	96	XM_006526668.3
*Amh*	F: 5′-TGCTAGTCCTACATCTGGCTGA-3′ R: 5′-GTCCAGGGTATAGCACTAACAGG-3′	120	XM_006513119.3
*Star*	F: 5′-GCTGCAGAAGCTCAACAACC-3′ R: 5′-TTGTCCCGTTCATCTGGTGG-3′	104	NM_010828.3
*Cyp19a1*	F: 5′-GAACAACCCTTGAGCACCTC-3′ R: 5′-AGCTTGGTGCCTTAATCCTTTC-3′	111	NM_011485.5
*Hsd3b1*	F: 5′-TTTTCGCTGAGAGACGTGGAG-3′ R: 5′-CCTCTGGATACTCTGCGACG-3′	135	NM_007810.4
*Lhcgr*	F: 5′-GCTGCACAGGAATAAAGGACA-3′ R: 5′-CATGCCTGCTTCGTGACCAT-3′	89	NM_008293.4
*Fshr*	F: 5′-AATGAGTCCATCACGCTGAAAC-3′ R: 5′-CCTGCAATTTGGTGGAAGAGA-3′	187	NM_001364898.1
*Bcl2*	F: 5′-AGTACCTGAACCGGCATCTG-3′ R: 5′-TATGCACCCAGAGTGATGCAG-3′	169	NM_009741.5
*Bax*	F: 5′-CCCGAGCTGATCAGAACCAT-3′ R: 5′-TTCCTAATGCCAACCTGTGAAG-3′	139	XM_011250780.2
*Caspase3*	F: 5′-GCTTGGAACGGTACGCTAAG-3′ R: 5′-CCACTGACTTGCTCCCATGT-3′	112	NM_001284409.1

## Data Availability

Data supporting the findings of this manuscript have been included in the manuscript. The other data will be made available on request.

## Abbreviations

IGF-1	Insulin-like growth factor-1
3D	Three-dimensional
AMH	Anti-Müllerian hormone
GCs	Granulosa cells
GDF-9	Growth and differentiation factor-9
BMP-15	Bone morphogenetic protein-15
FGF-8	Fibroblast growth factor-8
FSHR	Follicle Stimulating Hormone Receptor
LHCGR	Luteinizing Hormone/Choriogonadotropin Receptor
StAR	Steroidogenic Acute Regulatory Protein
HSD3B1	Hydroxy-Delta-5-Steroid Dehydrogenase, 3 Beta- And Steroid Delta-Isomerase 1
IGFBPs	Insulin-like growth factor-1 binding proteins
IGF-1R	Insulin-like growth factor-1 receptor
FBS	Fetal bovine serum
IVM	In vitro maturation
SPSS	Statistical Package for the Social Sciences
hCG	Human chorionic gonadotropin
eCG	Equine chorionic gonadotropin
